# Application of a Fork Plate for Greater Trochanter Osteosynthesis in Total Hip Arthroplasty

**DOI:** 10.17691/stm2020.12.2.10

**Published:** 2020

**Authors:** A.I. Avdeev, D.G. Parfeev, I.A. Voronkevich

**Affiliations:** PhD Student, Russian Scientific Research Institute of Traumatology and Orthopedics named after R.R. Vreden, 8 Academician Baikov St., Saint Petersburg, 195427, Russia;; Head of Traumatology and Orthopedics Unit No.1, Russian Scientific Research Institute of Traumatology and Orthopedics named after R.R. Vreden, 8 Academician Baikov St., Saint Petersburg, 195427, Russia;; Head of the Scientific Department for Treatment of Traumas and Their Sequelae, Russian Scientific Research Institute of Traumatology and Orthopedics named after R.R. Vreden, 8 Academician Baikov St., Saint Petersburg, 195427, Russia

**Keywords:** trochanter plate for osteosynthesis, osteosynthesis, greater trochanter fixation, arthroplasty.

## Abstract

**The aim of the study** was to assess the effectiveness of using an original technique of greater trochanter fragment fixation during total hip replacement.

**Materials and Methods:**

Since 2013, this construction has been implanted in 175 patients in the clinic of the Russian Scientific Research Institute of Traumatology and Orthopedics named after R.R. Vreden. By the present time, 50 patients have been examined in order to assess the effectiveness of this construction. They were divided into three groups depending on the cause of greater trochanter fixation by the original plate: osteotomy according to T. Paavilainen, periprosthetic Vancouver type AG fractures, false joints of the greater trochanter. The degree of consolidation of the greater trochanter fragment with the femoral bone metadiaphysis was assessed using the roentgenometric technique according to M. Hamadouche’s recommendations. The functional result of the hip was evaluated according to the Oxford Hip Score scale.

**Results:**

The nonunion rate in the presented groups was 8.3% in fragment fixation after osteotomy according to T. Paavilainen, 25% after fragment fixation in periprosthetic Vancouver type AG fracture, and 11.1% in fixation of the greater trochanter fragment when treating a false joint of the given localization.

**Conclusion:**

Preliminary results of the greater trochanter fixation using the original fork plate during total hip replacement showed sufficient effectiveness not only in osteotomies of the greater trochanter, periprosthetic iatrogenic and pathological Vancouver type AG fractures but also in case of false joints of this localization developed due to the failure of the previous fixation.

## Introduction

In Russia and in the world in general, a tendency to the increasing number of operations for total hip replacement has been noted [1]. Total hip arthroplasty finds its application in different complicated clinical situations, e.g. in dysplastic coxarthrosis with a high dislocation of the femoral head, in nonunion fractures of the trochanteric area as well as in various posttraumatic deformations [2, 3].

**Figure 1:**
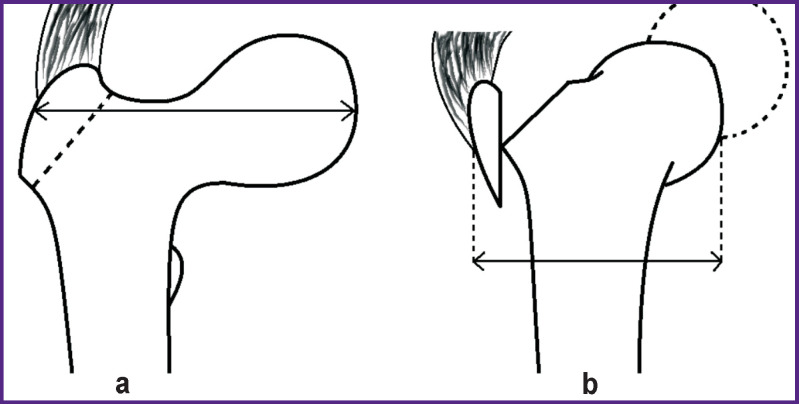
Proximal part of the femoral bone [4]: (а) before transposition (inverted L-shape); (b) after osteotomy and transposition of the greater trochanter fragment (Т-shape)

Still at the early stage of forming the concept of modern hip replacement operations, Charnley noted in his works that there is no alternative to trochanteric osteotomy for the correction of deformations in this anatomical area [4]. The author believes that it enables the restoration of the abductor lever arm length due to the changes in the form of the proximal part of the femoral bone, i.e. from the inverted — L-shape to the T-shape (Figure 1) that, in its turn, normalizes biomechanics of the entire hip [5].

Presently, the effectiveness of the offset restoration does not give rise to doubts in traumatologists-orthopedists, however, the problem of greater trochanter (GT) fixation has not yet been completely solved [6, 7].

The group of patients with periprosthetic fractures of GT attracts special attention. According to the observations presented by Korytkin et al. [3], absence of GT union during treatment of periprosthetic fractures of the femoral bone of the AG type with different variants of osteosynthesis were observed in 65% of cases. It speaks of the low effectiveness of GT fragment fixation methods for the given type of fractures.

Failure of GT fragment osteosynthesis results in the development of painful pseudoarthrosis, requiring a higher level of fixation reliability for its removal. During revision hip arthroplasty iatrogenic GT fractures occur rather often which may be associated with local osteoporosis, and in case of pathological lysis of the bone tissue these fractures may happen even spontaneously. All these damages also require rigid and long-functioning fixation of the GT fragment compelling to create more perfect methods and devices [7, 8].

**The aim of the study** was to assess the effectiveness of using an original technique of greater trochanter fragment fixation during total hip replacement.

## Materials and Methods

**Figure 2:**
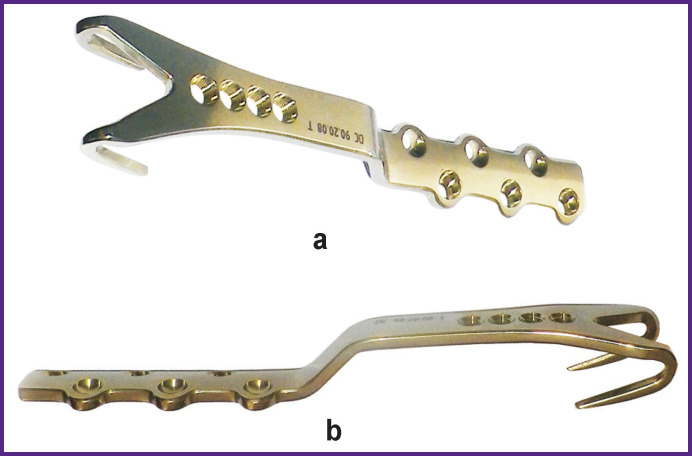
Original plate for fixing a greater trochanter fragment: (а) rear view; (b) side view

A special plate for GT fragment fixation (Figure 2) has been designed at the Russian Scientific Research Institute of Traumatology and Orthopedics named after R.R. Vreden [9].

The plate was manufactured in the form of a two-pronged fork. Prongs are 4 mm thick at the base and 30 mm long, they are oriented against the gluteal muscle traction. To minimize the mobility, there are four holes in the trochanteric part of the plate with an angular stability for the locking screws of 3.5 mm in diameter, AO/ASIF standard. The plate body of 5 сm standard length is provided with six holes for cortical screws of 3.5 mm in diameter with divergent channels typical for the periprosthetic plate.

**Figure 3:**
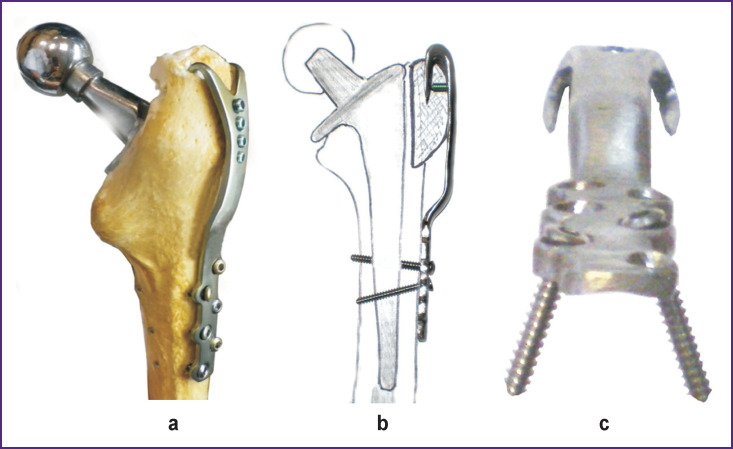
Fixator for greater trochanter of the hip: (а) view on the bone; (b) side view (scheme); (c) divergent screw orientation for intracortical fixation

Cortical screws are passing tangentially through a compact bone layer bypassing the endoprosthesis stem and are fixed by the thread in the bone or partially cut into the cementum mantle. This essentially increases stability of the diaphyseal fixation of the plate body (Figure 3).

When inserting the angle-stable screws in each of the same holes, they pass through the trochanter and are fixed monocortically in the underlying compact layer of the metaphysis acting as anchors. Their main task is to stabilize the proximal part of the plate to prevent shear loads. Thus, the fork blade works against “tearing-off” muscle traction, i.e. against the shear in the proximal direction, while the screws with angle stability work against rotary microshifts in any direction. The plate bending similar to the GT shape allows the bypass of its fragment along the contour [10] whereas tightening of the diaphyseal screws provides compression over the surface of the contact: pressing the trochanter to the femoral bone.

In the period from 2013 till present days, this construction has been implanted in 175 patients in the clinic of the Russian Scientific Research Institute of Traumatology and Orthopedics named after R.R. Vreden: to the patients with dysplastic coxarthrosis (osteotomy according to T. Paavilainen) [2], periprosthetic TG fractures (Vancouver AG) [3, 11, 12], and false joints in the area of the greater trochanter. In order to assess the effectiveness of the construction application, 50 patients have been examined by the present time. They were divided into three groups depending on the cause of greater trochanter fixation (Table 1).

**Table 1:** Patient characteristics

Characteristics	Group 1 — osteotomy according to T. Paavilainen (n=24)	Group 2 — periprosthetic fracture, Vancouver AG (n=8)	Group 3 — false joint of greater trochanter (n=18)
Age (years)	46.7	58.5	51.4
Gender (females/males)	19/5	8/0	14/4
Follow-up period (months)	15.2	10.5	12.5

The degree of consolidation of the GT fragment with the femoral bone metadiaphysis was assessed using the roentgenometric technique according to M. Hamadouche’s recommendations [6]. The functional result of the hip was evaluated according to the Oxford Hip Score scale.

The study complies with the Declaration of Helsinki (2013) and was approved by the Ethical Committee of the Russian Scientific Research Institute of Traumatology and Orthopedics named after R.R. Vreden. Written informed consent was obtained from all the patients.

**Statistical data processing.** Data were statistically processed using parametrical and nonparametric analysis. Accumulation, correction, and systematization of the initial information and visualization of the obtained results were performed using spreadsheets Microsoft Office 365. The program Past v. 3.25 was used for statistical analysis.

## Results

The obtained results of using the original plate in the examined groups are presented in Table 2. These functional results, as well as the rate of GT fragment nonunion, give evidence of the effectiveness of the suggested device in all groups. Statistically significant differences between the results in the group were absent.

**Table 2:** Results of original plate application

Indices	Group 1 — osteotomy according to T. Paavilainen (n=24)	Group 2 — periprosthetic fracture, Vancouver AG (n=8)	Group 3 — false joint of greater trochanter (n=18)	p
Oxford Hip Score (points), Me [Q1; Q3]	41 [38.5; 43]	38 [37; 40.5]	39.5 [36; 45.5]	0.368*
Consolidation of greater trochanter (abs. number/%):				
successful	22/91.7	6/75	16/88.9	
failure	2/8.3	2/25	2/11.1	0.450^+^

* Kruskal–Wallis Н test; ^+^ Pearson χ^2^ test.

Two complications (fractures of the endoprosthetic stem and plate) occurred in group 3 of our observation series. We consider them to be typical mistakes of adopting a new method. The plate broke due to its excess bending in the attempt to adjust it to the shape of the trochanter. Besides, surgeons tried to bend the plate body across the hole which resulted in the occurrence of stress concentrator and subsequent crack in the metal. This case shows that redundant deformation should be avoided especially its concentration on the hole during fixture modeling. If significant bending of the plate is necessary (in case of severe dysplasia and consequences of supportive osteotomies), these manipulations should be done before the operation at the stage of preparation which will allow for the application of a wider spectrum of instruments and avoidance of point deformation leading to metal fracture.

Fracture of endoprosthesis stem, as the revision operation showed, was caused by the stem damage with a drill due to the surgeon’s mistake. The drill left a marginal defect on the stem which resulted in the crack in the metal. It should be noted that consolidation of GT fixed with the given plate occurred timely in this patient, i.e. the construction has fulfilled its task. In this connection, surgeons should be recommended to be more attentive when forming the channels for the screws: to make them intracortically avoiding putting the drill into the channel and preventing the contact of the drill with the endoprosthesis stem.

Below is a clinical example of using the proposed fixator.

*A female patient S., 63 years of age, visited the clinic at Russian Scientific Research Institute of Traumatology and Orthopedics named after R.R. Vreden in December 2017 presenting the complaints of pain, limited movement amplitude in both coxofemoral joints, shortening of the lower limbs. She considered herself sick since childhood, she does not remember any traumas to occur. The patient’s body mass index was 31.56 (excess). On objective examination, marked hypotrophy of muscles in the area of the left hip was determined. Trendelenburg’s symptom was positive. Moderate contracture of the left hip was observed.*

**Figure 4:**
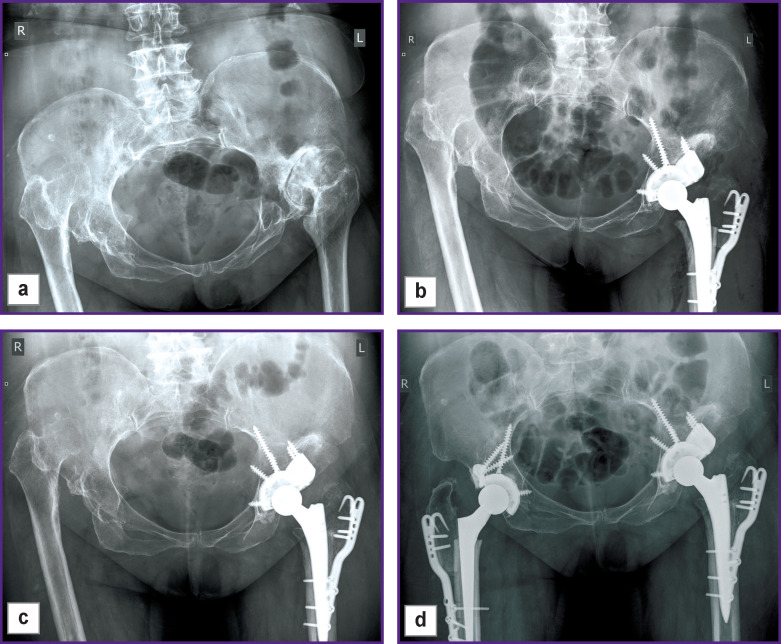
Clinical example of using the original fixator in patient S., 63 years old. Survey radiograph of the pelvis: (а) before the operation; (b) on the first day after total arthroplasty of the left hip; application of trochanteric osteotomy according to T. Paavilainen, fixation of the greater trochanter by the fork plate; (c) 6 months after the operation; radiographic signs of greater trochanter fragment consolidation with the hip are noted; (d) similar application of the method on the contralateral joint to eliminate the difference in the length of the lower limbs

*According to the radiographic examination of December 2017 before the operation (Figure 4 (a)) signs of bilateral dysplastic coxarthrosis, bilateral dislocation of the hip, C1 according to Hartofilakidis classification were found, Barnett–Nordin index was 0.552 (norm). After preliminary examination, the patient underwent total arthroplasty of the left hip with a shortening osteotomy of the GT according to T. Paavilainen and implantation of the individual acetabular component.*

*Radiographic examination on the first day after the operation (Figure 4 (b)): the state after total left hip replacement with shortening osteotomy according to T. Paavilainen, the position of the components is correct.*

*Six months after the operation: pain symptom in the area of the left hip is absent. Trendelenburg’s symptom is negative. According to radiography at that time (Figure 4 (c)): radiographic signs of consolidation of the GT fragment with the metadiaphysis of the left femoral bone, the state after total left hip arthroplasty, the position of the components is correct, signs of construction instability are absent. The patient’s score is 39 according to the Oxford Hip Score corresponding to a good functional result.*

*In June 2018 the patient underwent operation on the contralateral joint using a similar technique (Figure 4 (d)).*

## Discussion

Isolated fractures of GT occur rather seldom. Therefore, the development of the idea to fix the GT fragment started when approaches with different variants of trochanter osteotomies were required in complicated cases of arthroplasty [5]. The development of GT fixation methodology was going on rather intensively since osteosynthesis failure led to the formation of a GT false joint that greatly influenced the final result of treatment on the whole [6, 7, 13]. During the last 50 years, diverse constructions for GT fragment fixation have been proposed and the gained experience was taken into consideration when these methods were improved [2].

Today, one of the most common variants of GT fixation is an extramedullary plate-onlay with the application of cables developed by Dall and Miles [14]. In the work [15], the authors presented promising results of using this extramedullary plate-onlay (GT cable grip system): in 44 of 46 cases of osteosynthesis, there was complete consolidation of GT with the hip metadiaphysis. However, the drawback of all cerclage and cable systems is a quick loss of cable tension under which atrophy of the bone tissue develops due to pressure, and the more the cable is strained, the more intensive is the loss of the bone tissue [7]. The device like this can function during a limited period of time: till the bone lysis under the cable leading to the strain loss and consequently to fixation failure, and rotary movement, whereas a cyclic traction of the gluteal muscles destroys the forming callus between GT and the hip.

A certain role in solving this problem in recent years played the development of methods for fixing the hip fragments. Fernandez et al. [13] reported excellent results of using compressing trochanteric hook plate for treatment of false joints in the trochanteric area and used the original construction together with bone grafting.

In 2009 for the first time there appeared a report on the application of the plate with angular stability for GT fixation in total hip replacement. However, it was a condylar tibial plate with angular stability rather than a specially designed construction that was used for trochanter fragment fixation [10]. In 2016, the authors [16] reported the results of treating 32 patients. In 22 of them GT was fixed by a condylar plate for the tibia, and in 10 cases, a specially designed periprosthetic plate was used for the proximal part of the femoral bone. Fibrous union was observed in 6.2% of cases, complete absence of consolidation in 3.1%. The results presented show the necessity of developing and application of plates with the possibility of locking the screws for GT fixation during total hip replacement and in GT nonunion.

The device presented in the article possesses the construction elements and features proposed previously in the designs of hook and periprosthetic plates determining a new level of rigidity and reliability of GT fragment fixation.

## Conclusion

The results obtained in the course of studying the possibility of greater trochanter fixation with the original fork plate during total hip arthroplasty have shown sufficient effectiveness in greater trochanter osteotomies, periprosthetic iatrogenic and pathological Vancouver type AG fractures, and also in false joints of this localization developed due to the failure of the previous fixation. The failure analysis (8.3% of nonunions) revealed mistakes in the application of the new device which were typical for the period of adoption of new methods. Their critical assessment will allow us to find the way for improving the construction itself and the technology of its application.
